# Enhancing lane detection in autonomous vehicles with multi-armed bandit ensemble learning

**DOI:** 10.1038/s41598-025-86743-z

**Published:** 2025-01-25

**Authors:** J. Arun Pandian, Ramkumar Thirunavukarasu, L. Thanga Mariappan

**Affiliations:** School of Computer Science Engineering and Information Systems, Vellore Institute of Technology, Vellore, India

**Keywords:** Autonomous vehicles, Convolutional neural networks, Ensemble learning, Multi-armed bandit, Thompson sampling, Engineering, Mathematics and computing, Computational science, Computer science, Information technology, Software

## Abstract

This study introduces a novel ensemble learning technique namely Multi-Armed Bandit Ensemble (MAB-Ensemble), designed for lane detection in road images intended for autonomous vehicles. The foundation of the proposed MAB-Ensemble technique is inspired in terms of Multi-Armed bandit optimization to facilitate efficient model selection for lane segmentation. The benchmarking dataset namely TuSimple is used for training, validating and testing the proposed and existing lane detection techniques. Convolutional Neural Networks (CNNs) architecture which includes ENet, PINet, ResNet-50, ResNet-101, SqueezeNet, and VGG16Net are employed in lane detection problems to construct segmentation models and demonstrate proficiency in distinct road conditions. However, the proposed MAB-Ensemble technique overcomes the limitations of individual models by dynamically selecting the most suitable CNN model based on prevailing environmental factors. The proposed technique optimizes the segmentation accuracy and treats the attained accuracy as a reward signal in the context of reinforcement learning by interacting with the environment through CNN model selection. The MAB-Ensemble achieved an overall accuracy of 90.28% in different road conditions. The results overcome the performance of the individual CNN models and state-of-the-art ensemble techniques. Also, it demonstrates superior performance which includes daytime, night-time, and abnormal road conditions. The MAB-Ensemble technique offers a promising solution for robust lane detection by harnessing the collective strengths of diverse CNN models.

## Introduction

Urban Traffic Congestion (UTC) is a pervasive problem in cities worldwide. The UTC significantly impacts the quality of life, economic productivity, and environmental sustainability^1^. The UTC arises from factors including the increased number of vehicles, inadequate infrastructure, commuting patterns, and environmental factors. Autonomous vehicles have already been employed in the transportation systems of most developed countries. Autonomous vehicles have the potential to reduce the UTC by controlling the factors. Autonomous vehicles can increase travel efficiency and safety. Also, they reduce traffic congestion and environmental impact^2^. Assessing traffic situations, recognizing obstacles, accurate lane detection, and performing flawless and real-time communication with the objects in the network are some of the forefront research avenues in this emerging field^3^. An accurate lane detection system is an integral component that would offer intelligent real-time provision to the vehicle in deciding the right path based on the geometrical features of the lane line to fully comprehend the driving environment with a large number of road users. Further, the system also enables the vehicle to stay in the assigned lane and avoid colliding with vehicles in other lanes. Some environmental factors and technical challenges are the reasons behind the faulty detection of lanes by autonomous vehicles^4^. Environmental factors such as lighting conditions, road conditions, shadows, and reflections influence automatic lane detection. The technical challenges include computational power, sensor limitations, and complexity of algorithms used.

Most traditional methods of traffic line detection techniques extract low-level traffic lines using hand-crafted features, which include colour^5,6^or edges^7,8^. Such low-level features have been combined using a Hough transform^9,10^or Kalman filter^11^to generate traffic line segment information. The traditional techniques are simple to adopt and computationally inexpensive. However, the performance of the traditional technique is inadequate in different environmental conditions such as lighting, shadows, and reflections^12^. Also, the techniques are not efficiently detecting the lane lines in road intersections and poor road conditions. Hence, the traditional lane detection methods are not sufficient for an autonomous driving environment.

The proliferation of artificial intelligence has been greatly witnessed due to the emergence of well-performing deep learning models. In the context of computer vision, deep learning models have been employed to omit hand-crafted features and learn to extract features in an end-to-end manner^13,14^. Convolutional Neural Networks (CNNs) are deep learning techniques that outperform traditional approaches in computer vision applications^15–17^. Thus, these models exhibit improved performance in automated feature extraction, classification, and detection of objects, which are highly essential for fulfilling the real-time requirements of computer vision applications^18,19^. Also, the CNNs are implemented in various applications on autonomous vehicles including security, obstacle detection, road sign recognition, driver monitoring, and traffic signal recognition^20^. The practical implementation of an accurate lane detection system still needs further exploration despite the evaluation of the CNN models^21^. This is due to the inherent slenderness of the road, the complex and changing nature of road conditions, and the existence of a wide variety of lane markers. Though lane detection algorithms based on the CNN models alleviate the detection problem caused by environmental changes, they still require improved solutions. Several CNN models have been developed for detecting the road lines. However, not all the CNN models performed well in all the environmental conditions. For instance, the PINet performed better in the daytime than in other environmental conditions.

In the context of machine learning, ensemble approaches produce viable solutions to combine the uniqueness of the individual machine learning models and aggregate the overall accuracy of the classifications, object detections, and segmentations^22^. However, ensemble approaches including majority voting, random forest, stacking and weighted averaging are ideal techniques in the standard classification task, where characteristics of the models are highly pre-declared. In a typical lane detection task, environmental parameters vary dynamically, and hence the utilization of existing ensemble approaches is inadequate to perform lane segmentation effectively^23^. Reinforcement Learning (RL) techniques are reactive learning techniques in machine learning that interact with the environment to learn the decision boundary effectively^24^. The RL models are implemented to achieve improved performance in various decision-making problems under dynamically changing environments^25^.

This research work aims to alleviate the advantages of the ensemble approach and the reinforcement learning technique in the lane detection task to improve accuracy. The lane detection problem has emerged as a single-state decision-making problem. A multi-armed bandit optimization technique offers significant advantages for solving single-state decision problems. This reinforcement learning approach allows for the dynamic selection of the most efficient CNN model under varying environmental conditions and road scenarios. The widely used and successful CNN architectures such as ENet, PINet, ResNet-50, ResNet-101, SqueezeNet, and VGG16Net, were selected as base learners in this study due to their proven effectiveness in lane segmentation tasks. The CNN models were separately trained on the TuSimple lane segmentation dataset. The RL agent was designed to control the ensemble process of the CNN models and dynamically select a suitable model for lane detection based on the current environmental factors. The RL agent ensures optimal performance in lane detection tasks by leveraging the strengths of these pre-trained architectures and combining them adaptively. This research demonstrates the advantages of employing an RL-driven ensemble approach to address the challenges of lane detection across diverse road scenarios, showcasing the synergy between reinforcement learning and CNN-based models.

The major contributions of this research are as follows:

(i) Proposed a Multi-Armed Bandit ensemble framework employing reinforcement learning to dynamically select the appropriate CNN models to fit into the different environments and road conditions.
(ii) Used popular CNN architectures (ENet, PINet, ResNet-50, ResNet-101, SqueezeNet, and VGG16Net) as base learners to capture the individual advantage of each CNN for lane segmentation tasks.
(iii) Implemented an RL agent that dynamically selects the best-fitted CNN model considering the current state of the road and the environment.
(iv) MAB-Ensemble was shown to be effective for lane detection through intensive experiments and comparisons with individual CNNs and other ensemble techniques.
(v) Solved real-time computational challenges through the feasibility of dynamic model selection for practical implementations in autonomous vehicle systems.
(vi) Proved that the MAB-Ensemble framework can adapt to future versions of CNN architectures; thus, it can be generalized and deployed in practical applications like autonomous driving.

The rest of the paper is organized as follows: Sect. 2 of the paper deliberates the state-of-the-art CNN models and their impact on lane detection tasks. The CNN model selection for lane detection, and the design and development of the proposed ensemble learning based on the Multi-Armed Bandit optimization approach, are presented in Sect. 3. A detailed experimental investigation of the proposed technique on the benchmarking dataset, namely the TuSimple lane detection dataset, is presented in Sect. 4. Concluding remarks and further scope are outlined in Sect. 5 of the paper.

## Related work

The CNN models are a popular technique for developing computer vision applications such as image classification, object detection, and segmentation. Lane detection on roads is a common image segmentation problem that has been efficiently solved using CNN models. However, there are some challenges and limitations in the existing CNN-based approaches for lane detection tasks. This literature survey discusses the most popular and efficient lane detection techniques using CNNs and their limitations.

In^26^, the first CNN-based lane detection approach used convolutional layers to extract lane features based on region of interest selection and boundary detection. Further, the random sample consensus technique was applied to cluster the extracted lane features. This approach detected lanes only in minimally complex road conditions. A DeepLanes technique was proposed for deep classification of lane features using the location information of the vehicle^27^, which restricts the application scenario of the proposed model. The technique did not focus on diverse road conditions. A Vanishing Point Gradient network (VPG) was proposed by the authors in^28^. The vanishing points in the network were used to guide lane line detection. However, the complex post-processing work of VPG requires high computational power.

In^29^, the authors extensively reviewed deep learning-based lane detection approaches and categorized them into three perspectives: classification methods, where prior information about lane position is used to discriminate the road boundary type^30,31^; object detection-based methods, where feature points of each lane segment are used as inputs for detecting the lane^32,33^; and segmentation-based methods, where pixels of input images are classified into individual classes^34–38^. All these approaches use deep learning-based feature extraction. They also discussed the limitations of each technique, such as computational complexity, adaptability issues with the dynamic nature of roads, and challenges related to climate and lighting conditions. On the other hand, pre-trained deep neural networks are used to achieve good performance with a smaller amount of data, optimizing the deep learning models^39^. The first few layers of such pre-trained models are used to recognize generic features and subsequent layers are modified to improve performance. However, these pre-trained models detect lanes effectively only under specific road conditions.

A fast and compact encoder-decoder-based neural network architecture, namely Efficient Neural Network architecture (E-Net), was proposed by the authors in^40^ for performing semantic segmentation of lane images. This technique is highly useful for autonomous vehicles. A larger encoder was used to handle lower-resolution data and for filtering purposes, whereas a smaller decoder was used to fine-tune the results. Each E-Net block consists of three convolutional layers, with batch normalization and the activation function, Parametric Rectified Linear Unit (PReLU), applied to each convolution. The performance of the E-Net network was validated on the benchmark TuSimple dataset, and the results showed that E-Net performed particularly well under daytime road conditions.

An effective attention distillation approach, namely Self Attention Distillation (SAD), was proposed in^41^ to improve the representation learning of CNN-based lane detection models. The attention maps of previous layers in the network are useful for providing contextual information to successive layers. In the existing E-Net, a small network was added to predict the existence of lanes. Additionally, dilated convolution was used to replace the original convolution layers without increasing the number of parameters. Experimental results show that this approach improves the visual attention performance across different network layers, including ResNet-18, ResNet-34, and ResNet-101. Among the different ResNets, ResNet-101 achieves the highest performance in lane detection.

A deep neural network based on CNN for solving the lane detection problem using second-order polynomial coefficients was developed in^42^ to detect road lines. This approach fused the existing ResNet-50 layers with fully connected layers. The first two fully connected layers are used after the dropout layer, while the remaining six fully connected layers estimate the lane coefficients for each line to fit a second-order polynomial. The input image size was reduced to 160 × 160, and lane line points were scaled to obtain the target coefficients for the polynomial. Results were evaluated using the mean squared error loss function and the RMSprop optimizer. Higher accuracy was achieved by using the ResNet-50 architecture with a batch size of 32. However, this approach processes only low-resolution images.

In^43^, a lightweight CNN model, namely Squeezenet, was proposed for the lane detection task. An experimental dataset comprising 2,770 images was used, which includes images under different conditions such as normal illumination, insufficient illumination, and ground reflection. The model uses a hybrid convolution kernel of 1 × 1 and 3 × 3 sizes in Squeezenet and is applied to the intelligent car tracking task. The Squeezenet network achieved both good accuracy and speed when compared to the existing lane classification technique, where ResNet-34 was used as the backbone network.

In^44^, the authors proposed a Point Instance Network (PINet) for lane detection. The idea of a stacked hourglass network was adopted in their work for key points estimation and object detection. The PINet consists of a resizing network, a predicting network, and an output branch. Input data of size 512 × 256 is compressed into a smaller size of 64 × 32 by the resizing network and forwarded to the predicting network, which contains several hourglass modules in series for feature extraction. The final output branch predicts the exact location of traffic line points from the extracted features. Due to the construction of the hourglass module with the same output branch, the PINet can be modified according to the computing power requirements. However, PINet has limitations when identifying road marks in small areas.

The authors in^45^attempted to compare six different CNN architectures, namely AlexNet, SqueezeNet, ResNet-18, ResNet-50, VGG16, and NASNet, for the lane detection problem under different adverse conditions, including harsh weather, illumination variations, and shadows. They customized the last layer of the pre-trained networks without affecting the rest of the layers. The ADAM optimizer was used to fine-tune the network parameters. The pre-existing networks were applied to both normal daytime driving conditions and challenging situations. It was observed that all networks struggled to attain good performance, except under normal daytime conditions. Among all the networks, VGG16 produced consistent performance inTuSimple^46^ dataset.

Ensemble learning techniques combine multiple base learners to improve the accuracy and robustness of predictive models. Majority Voting, Random Forest, Stacking, and Weighted Averaging are some widely used ensemble methods. Majority Voting is a simple technique where the final prediction is made based on the majority of predictions from individual base models^47^. However, it assumes that all models contribute equally to the final decision and fail to adapt to changing conditions. This can lead to suboptimal performance in dynamic environments. Random Forest builds an ensemble of decision trees using random subsets of the training data^48^. It aggregates the predictions of these trees, but like Majority Voting, it does not adjust to changes in the data distribution over time. Random Forest can also be computationally expensive, especially with a large number of trees. Stacking involves training multiple base models and using their outputs as input to a second-level model, or meta-model, which learns to combine these predictions^49^. While stacking can improve performance by leveraging diverse models, it requires additional computational resources and is prone to overfitting if the base models are not diverse enough. Weighted Averaging combines the predictions of multiple models using predefined weights, with better-performing models receiving higher weights^50^. While it is effective in regression tasks, it assumes a linear contribution from each model, which may not be suitable for complex, non-linear relationships. Like Majority Voting, the weights are fixed and do not change during deployment, limiting adaptability.

The extensive review emphasizes the importance of CNN-based deep learning models in achieving good accuracy in lane detection tasks. It discusses some of the most popular CNN models in this domain, such as ENet, PINet, ResNet-50, ResNet-101, SqueezeNet, and VGG16Net, while also addressing the advantages and limitations of these existing models. Despite the good performance of current state-of-the-art CNN models in specific road and environmental conditions, no single model performs well across all possible conditions. Additionally, the limitations of traditional ensemble techniques highlight the need for adaptive methods that can dynamically adjust to changing conditions. In real-world applications, such as autonomous driving, environmental factors like lighting, weather, or road conditions can vary rapidly, affecting model performance. Traditional methods are static and cannot adapt to these variations.

## Proposed work

In this section, a novel methodology is outlined to enhance lane detection for autonomous vehicles. The approach commences with meticulous collection and preparation of a diverse dataset representative of real-world road scenarios. This dataset serves as the basis for training and evaluating the effectiveness of the proposed lane detection system. Subsequently, a thorough evaluation of various Convolutional Neural Network (CNN) models is conducted to identify the most suitable architecture for the task. This process involves assessing each model’s performance on the prepared dataset to ensure that the selected CNNs exhibit superior lane detection capabilities. Furthermore, an innovative ensemble technique is introduced to further strengthen the lane detection system. By Leveraging the K-arm bandit optimization with Thompson sampling, the outputs of the chosen CNNs are intelligently combined to create a robust and accurate lane detection ensemble model. By integrating diverse model outputs, the ensemble method enhances the system’s ability in different environmental conditions and improves the overall performance. This comprehensive approach represents a significant advancement in enhancing the reliability and safety of autonomous driving technologies.

### Data Collection and Preparation

The research employs the TuSimple dataset^46^, widely acknowledged as a leading benchmark for lane detection tasks, comprising 6,408 road images captured along US highways. Each image boasts a resolution of 1280 × 720 pixels, ensuring sufficient detail for accurate analysis. This dataset is meticulously curated, with 3,626 images allocated for training, 358 for validation, and 2,782 for testing, collectively known as the TuSimple test set. The TuSimple dataset, comprising 6,766 road images across training, validation, and testing sets, underwent a redistribution process. This redistribution involved combining and reassigning images to training, validation, and testing sets to allocate more data for the training process compared to testing.

**Fig. 1:**
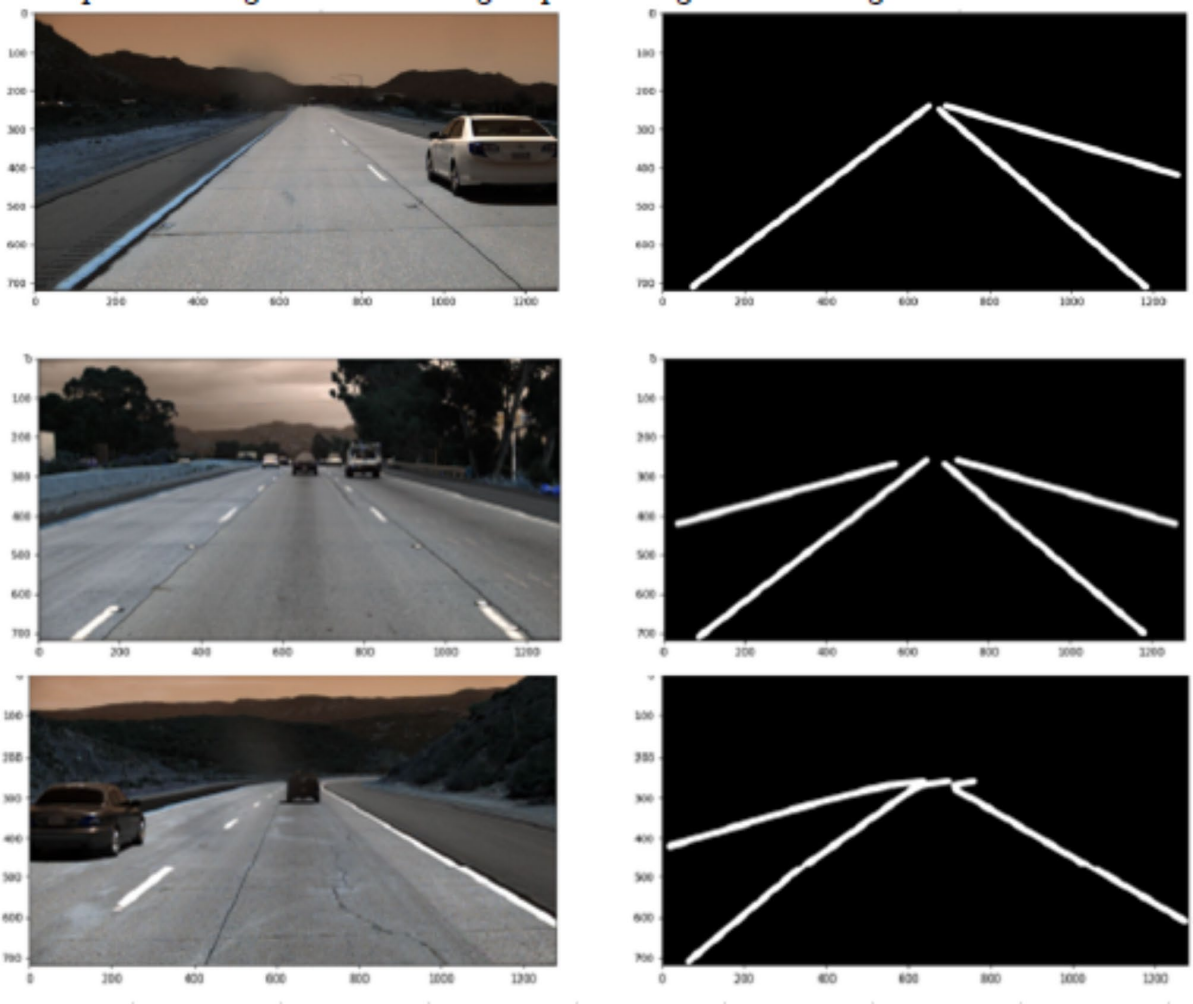
Sample colour and ground-truth images from the TuSimple dataset.

Adhering to the principle of providing ample training data for model learning, a distribution ratio of 80:10:10 was employed, allocating a larger portion to training. This step aimed to enhance the performance of both training and evaluation processes for proposed and existing segmentation approaches. By ensuring a balanced distribution of samples across the training, validation, and testing sets, the newly organized dataset facilitated more effective model training, validation, and testing. Consequently, the training set comprised 5,412 images, while both the validation and testing sets consisted of 667 images each. This standardized approach to dataset organization and distribution optimized the effectiveness and reliability of the experimentation process, thereby yielding more accurate and generalizable results for the evaluated segmentation models. Notably, the test set encompasses diverse weather conditions to ensure the model’s robustness across varying environmental contexts. Road images within the dataset are organized systematically within the “clips” directory, facilitating efficient data management. Accompanying label annotations are stored in JSON files corresponding to each clip, providing precise lane markings and other relevant information crucial for model training and evaluation. To facilitate model training, ground truth mask images are generated for each image in the training, validation, and test sets using the label annotations stored in the JSON files. This process enables the algorithm to learn from accurate representations of lane markings and other road features. Furthermore, the proposed work introduces a novel categorization scheme for the test dataset based on weather and road conditions. Images are classified into three distinct categories: “daytime,” - comprising images captured under bright lighting conditions; “nighttime,” -encompassing low-light scenarios; and “abnormal road conditions,” which includes images depicting challenging scenarios such as snow-covered roads, blurred scenes, lane intersections, and unusual lane configurations. The ratios of daytime, nighttime, and abnormal road condition images are 46%, 33%, and 21%, respectively. To facilitate the creation of ground truth mask images, the study leverages the capabilities of the OpenCV tool, renowned for its effectiveness in image processing tasks. This ensures the accuracy and efficiency of the mask generation process, allowing for seamless integration into the model training pipeline. Figure 1 presents illustrative examples of original road images providing visual insight into the dataset.

The ensuing subsection delves into an analysis of the efficacy exhibited by established transfer learning Convolutional Neural Network (CNN) architectures when applied to the TuSimple dataset for the task of lane segmentation.

### CNN Model Selection & Training

A segmentation model for lane detection from road images was developed by selecting a range of prominent CNN architectures: ENet, PINet, ResNet-50, ResNet-101, SqueezeNet, and VGG16Net. These models were chosen based on an extensive literature review. While traditionally used for classification tasks, they were adapted here for lane segmentation. Specifically, the classification layer in each model, typically featuring softmax activation, was modified. Instead of classification, this layer was replaced with three fully connected layers, transforming the models from classification-focused to regression-focused, in line with the lane segmentation task requirements. By substituting the softmax activation function with a regression-based approach, the models were optimized to predict continuous values, essential for accurately delineating lane boundaries in road images.

This strategic modification allowed the models to learn and predict the intricate spatial characteristics of lanes, enabling precise segmentation. By leveraging these state-of-the-art CNN architectures and customizing their output layers to suit the segmentation task, the proposed research work aims to enhance the accuracy and robustness of lane detection systems. This approach capitalizes on the wealth of features learned by the pre-trained models while ensuring their alignment with the specific demands of lane segmentation. Through meticulous adaptation and fine-tuning, the selected models are poised to offer superior performance in detecting and delineating lanes, thereby advancing the capabilities of autonomous driving systems and computer vision applications.

Each of the selected models was individually trained on the TuSimple dataset’s training subset, which consists of 3,626 images. The training process involved meticulous hyperparameter tuning using a random search technique to optimize model performance. The optimized hyperparameter values for each CNN model are provided in Table 1.

**Table 1:** Optimized hyperparameters for CNN models.

Hyperparameter	ENet	PINet	ResNet-50	ResNet-101	SqueezeNet	VGG16Net
Learning Rate	0.001	0.0005	0.0001	0.0001	0.001	0.0005
Batch Size	16	32	64	32	16	32
Optimizer	Adam	AdamW	SGD	SGD	Adam	AdamW
Dropout Rate	0.3	0.4	0.5	0.4	0.3	0.5
Weight Initialization	Xavier	Kaiming	Kaiming	Xavier	Xavier	He Normal
Data Augmentation	Yes	Yes	Yes	Yes	Yes	Yes
Regularization (L2)	0.0001	0.0005	0.001	0.0001	0.0001	0.0005

**Fig. 2:**
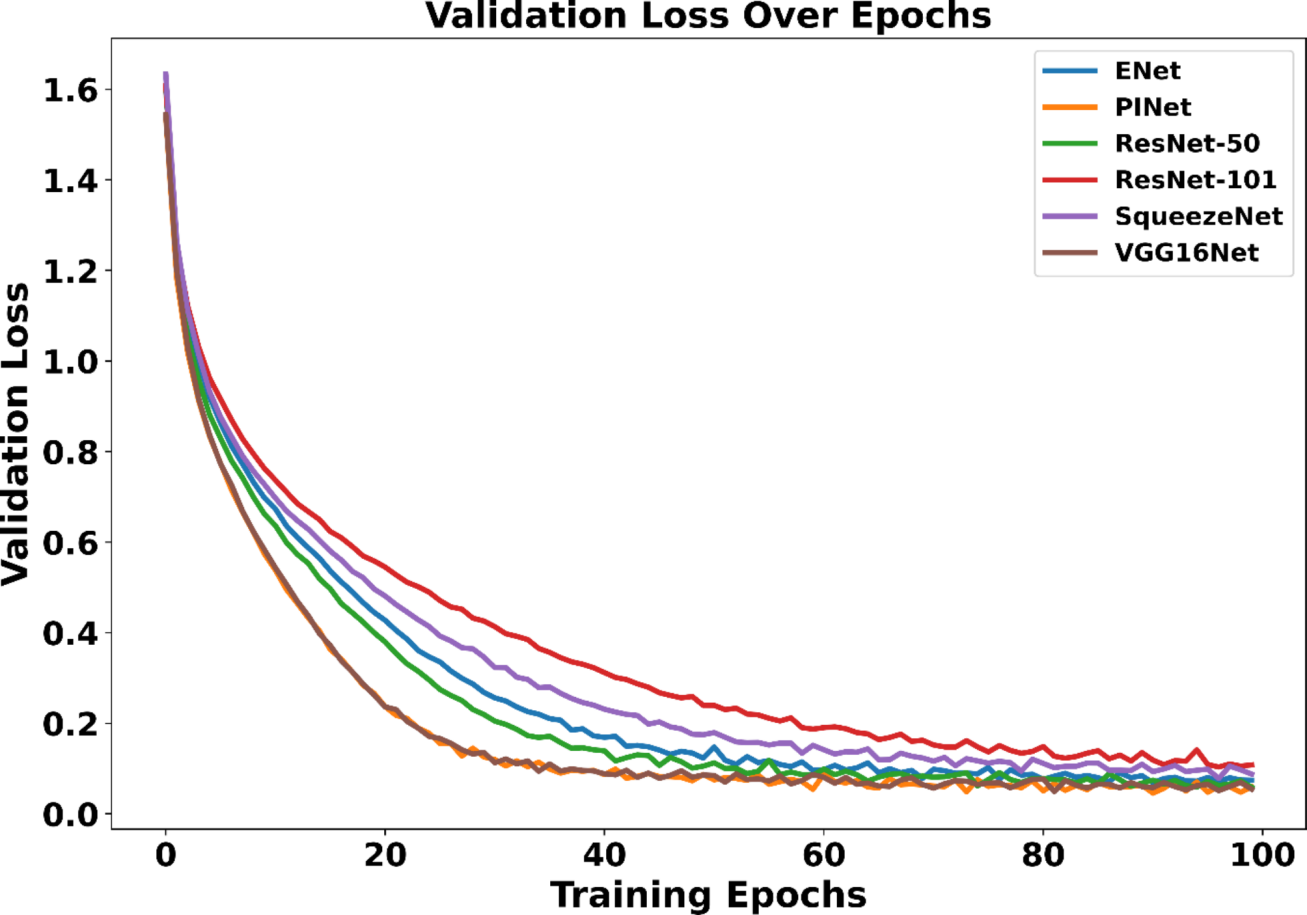
Validation performance of the selected models.

Training sessions of the ENet, PINet, ResNet-50, ResNet-101, SqueezeNet, and VGG16Net extended up to 100 epochs, executed on the Nvidia A100 GPU platform to expedite computations. Notably, the models demonstrated commendable performance, achieving validation losses below the threshold of 0.2 on the validation set in the TuSimple dataset. Figure 2 shows the validation loss of the CNNs in the validation data during the training process.

This discerning evaluation process underscores the efficacy of certain models over others, offering valuable insights into their suitability for accurate lane detection and segmentation, which is crucial for advancing autonomous driving systems and computer vision applications. Following the training phase, the selected models underwent rigorous testing on the TuSimple dataset’s test subset. Impressively, the overall performance of these models exhibited high accuracy levels, as detailed below: ENet achieved 79.9%, PINet attained 79.57%, ResNet101 produced 76.61%, ResNet50 demonstrated 75.1%, SqueezeNet delivered 79.2%, and VGG16Net yielded 76.27% accuracy. However, it became evident that the models’ performances varied across different road conditions. For instance, during daytime conditions, PINet outperformed other models, whereas SqueezeNet excelled in night-time settings. VGG16Net showcased superior accuracy in abnormal road conditions. Conversely, ResNet101 and ResNet50 exhibited average performance across all scenarios. Notably, while ENet emerged as the top performer overall, no single model consistently achieved maximum accuracy across all road conditions. This observation raises concerns regarding the deployment of a singular model for diverse real-world scenarios, as there’s a risk of diminished performance in certain conditions.

The comprehensive evaluation of each model’s performance under varying road conditions, including daytime, nighttime, and abnormal scenarios, was meticulously detailed in the results and discussion section. In addition to accuracy metrics, the Root Mean Square Error (RMSE), Intersection over Union (IoU) and Dice Coefficient were employed to provide further insights into model performance. The detailed analysis comprehensively presents the strengths and weaknesses of each model, offering valuable guidance for potential deployment in real-world applications. The findings prompt the importance of considering diverse environmental factors when developing and deploying lane segmentation models for autonomous driving and computer vision systems. While certain models may excel under specific conditions, achieving robust performance across all scenarios remains a challenge. Consequently, a holistic approach that integrates insights from various models and adapts to dynamic conditions is highly essential to ensure reliable performance in real-world settings. Further research and development efforts are warranted to address these challenges and enhance the resilience of lane segmentation systems in diverse road conditions.

In addressing the challenge of achieving robust lane segmentation across diverse road conditions, two potential solutions present themselves. The first entails seeking an alternative segmentation model capable of performing well under all environmental circumstances. However, this approach is not only financially burdensome but also fraught with challenges, as there remains a risk of encountering similar performance limitations. Conversely, the second solution, employing ensemble techniques, emerges as a more pragmatic and cost-effective strategy. Ensemble methods offer the advantage of harnessing the unique strengths of individual models, mitigating the need to search for a single, universally adept model. This approach capitalizes on the diverse characteristics and advantages of various models, thus enhancing overall performance. Ensemble learning techniques encompass a range of methodologies designed to integrate the predictions of multiple models. Common ensemble techniques include Bagging (Bootstrap Aggregating), Boosting (e.g., AdaBoost, Gradient Boosting Machines), Stacking, Random Forests, Ensemble of Neural Networks, and Bayesian Model Averaging. However, the challenge lies in devising an ensemble technique that can effectively discern and adapt to environmental conditions to make informed decisions regarding lane detection. Thus, there is a pressing need for the development of a novel ensemble technique tailored specifically to address the nuances of diverse road conditions.

The subsequent subsection of this research delves into the architecture and implementation process of a proposed novel ensemble technique for lane detection. By leveraging insights from existing ensemble methodologies and incorporating mechanisms for environmental awareness and adaptation, this novel approach aims to offer a comprehensive solution to the challenge of achieving reliable lane segmentation across varied road conditions.

### Ensemble CNN using multi-armed bandit optimization

To achieve a robust lane segmentation system on diverse road conditions, the proposed research introduces an innovative ensemble technique named the MAB-Ensemble, which utilizes Multi-Armed Bandit (MAB) optimization with Thompson sampling. The rationale behind this approach is to develop a learning methodology that is capable of making decisions based on environmental conditions with maximal rewards, aligning with the objectives of reinforcement learning. Reinforcement learning offers a framework for learning optimal actions through interaction with an environment to maximize cumulative rewards. By considering the same, the proposed ensemble technique aims to dynamically select the most suitable CNN model for lane detection based on the prevailing environmental conditions. The choice of MAB optimization is apt for this problem due to its relevance in single-state decision-making scenarios, where uncertainty, or randomness, prevails. In this context, the environmental conditions serve as the source of randomness, influencing the selection of the appropriate CNN model for lane detection. The MAB framework treats each CNN model as an “arm,” and the agent learns through exploration and exploitation to maximize rewards. Exploration involves selecting new possible CNN models to adapt to changing environmental conditions, while exploitation entails leveraging known optimal actions (CNN models) to maximize rewards.

**Fig. 3:**
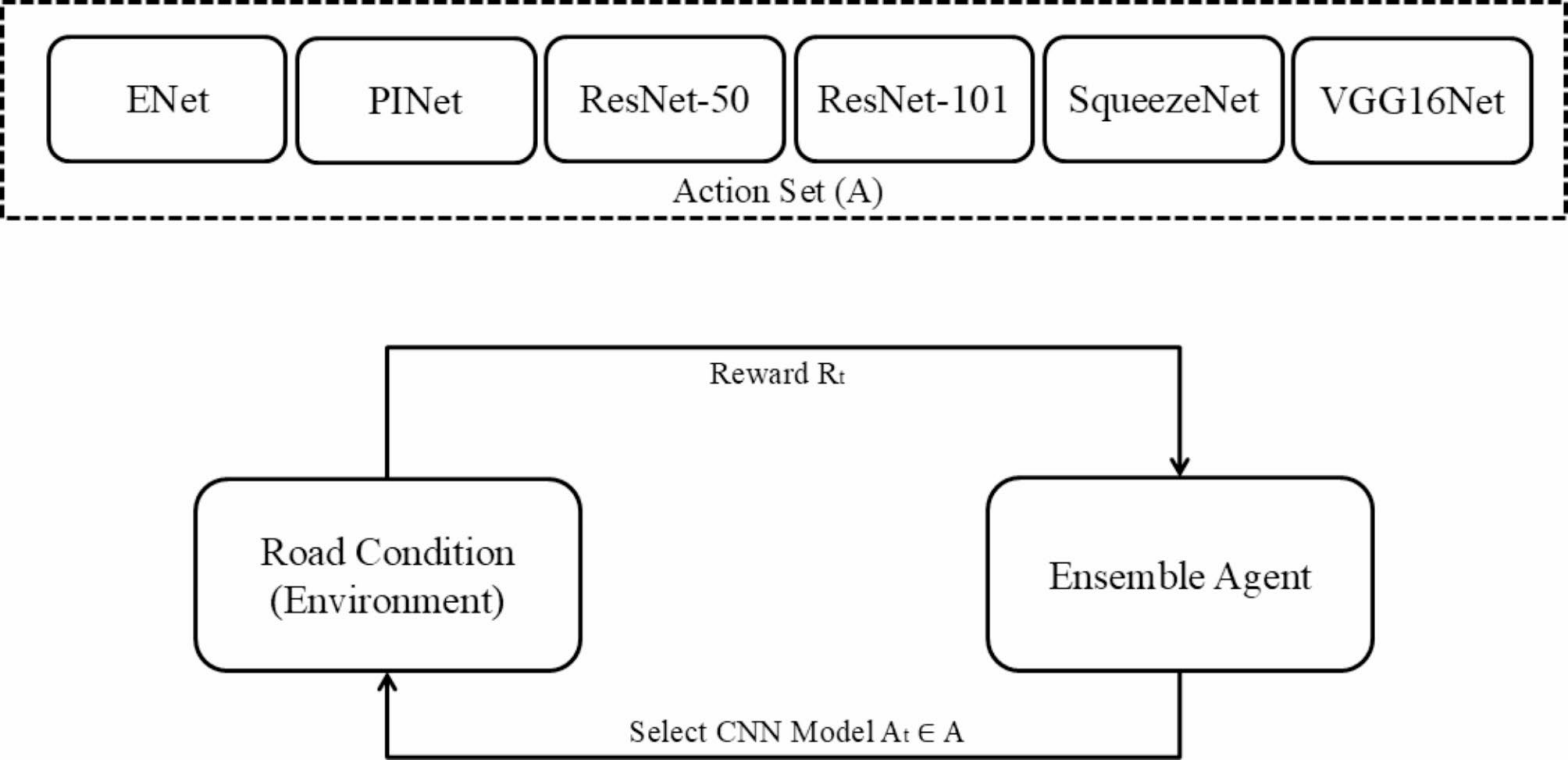
Architecture of the proposed MAB-Ensemble technique.

To address the exploration-exploitation trade-off inherent in MAB optimization, Thompson sampling—a Bayesian approach—is employed. Thompson sampling provides a principled method for the probabilistic selection of actions based on their estimated rewards to balance the exploration and exploitation problem. By incorporating Thompson sampling into the ensemble technique, the proposed approach adapts to changing environmental conditions, continuously learning and improving its performance over time. Figure 3 depicts the architecture of the proposed ensemble technique.

Mathematically, the MAB framework can be represented as follows:

Let NN denote the number of arms (CNN models), with N is 6 in this context. The ensemble model selects an action i at time step t based on Thompson sampling:

1 $$\:{\text{A}}_{\text{t}}=\:{\text{a}\text{r}\text{g}\text{m}\text{a}\text{x}}_{\text{i}}\:\left({{\uptheta\:}}_{\text{i},\text{t}}\right)$$

Where $\:{{\uptheta\:}}_{\text{i},\text{t}}\:$represents the estimated reward distribution for arm i at time step t, updated using Bayesian inference. The ensemble model receives a reward R_t_ based on the performance of the selected action:

2 $$\:{\text{R}}_{\text{t}}=\:\left\{\begin{array}{c}+1\:\:\:\:\:\:\:\:\:\:\:\:if\:action\:{\text{A}}_{\text{t}}\:is\:optimal\\\:-1\:\:\:\:\:\:\:\:\:\:\:\:Otherwise\:\:\:\:\:\:\:\:\:\:\:\:\:\:\:\:\:\:\:\:\:\:\:\:\:\end{array}\right.$$

The ensemble model learns to maximize cumulative rewards through iterative training and refinement. It improves lane segmentation performance across diverse road conditions. In this context, positive rewards (+ 1) are assigned for selecting the best action (optimal CNN model), while negative rewards (−1) denote sub-optimal choices. The binary reward system (e.g., + 1 for the highest-performing model and − 1 for others) is designed to emphasize the selection of the most suitable model under the current environmental and road conditions. This approach ensures faster convergence and reduces ambiguity during the training process. A continuous reward system was not considered as it introduces challenges, such as creating a gradient of rewards that complicates model selection, particularly under non-stationary conditions, where relative differences in performance matter more than absolute accuracy values. The ensemble model learns to adapt to varying environmental conditions through iterative training and exploration. It maximizes the rewards and improves lane segmentation performance.

The MAB-Ensemble technique is sensitive to its hyperparameters. These hyperparameters were optimized using the random search hyperparameter tuning technique. The cumulative reward was used as the evaluation metric to assess the effectiveness of each hyperparameter combination. The combination that produced the maximum cumulative reward was selected to train the MAB-Ensemble technique. Table 2 shows a list of the tuned hyperparameters and their optimized values.

**Table 2:** Optimized hyperparameters for the MAB-Ensemble technique.

Hyperparameter	Value
Learning Rate (α)	0.01
Discount Factor (γ)	0.9
Reward System	Binary
Maximum Episodes	1000
Maximum Steps per Episode	500
Exploration Strategy	Thompson sampling

**Fig. 4:**
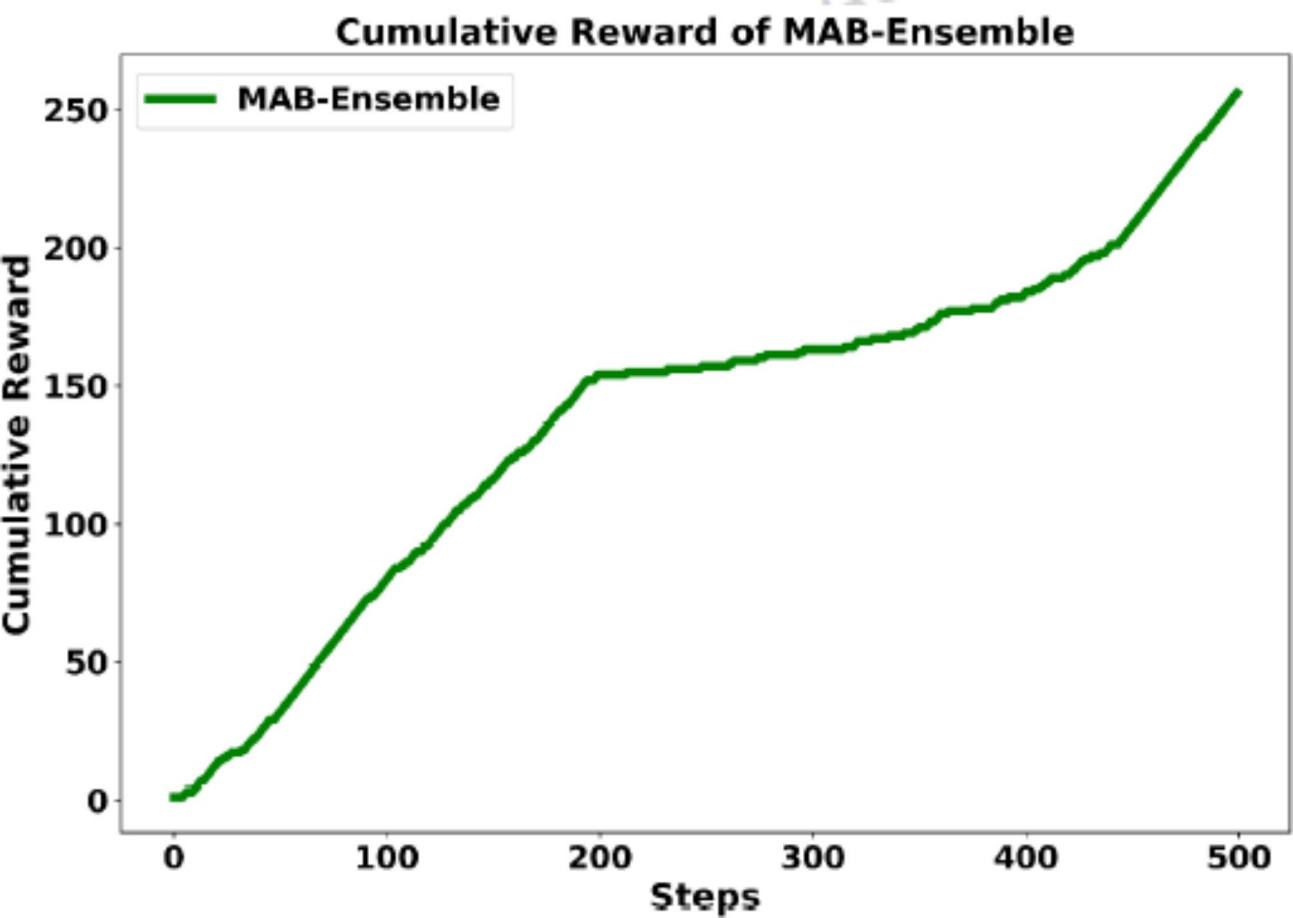
Cumulative reward achieved by the MAB-Ensemble technique.

The ensemble model is trained on the training dataset using the trained CNN models as actions, with iterations conducted over 500 steps and 1,000 runs to maximize the performance. Figure 4 illustrates the average reward achieved by the proposed ensemble learning approach over the number of steps.

In the subsequent section of the paper, the performance of the proposed ensemble technique on the test dataset is evaluated and compared against individual CNN models such as ENet, PINet, ResNet-50, ResNet-101, SqueezeNet, and VGG16Net using standard performance metrics. The standard performance metrics are segmentation accuracy, RMSE, IoU and Dice Coefficient. This comparative analysis provides insights into the efficacy of the proposed ensemble approach in handling diverse road conditions and its superiority over individual models.

## Results & discussion

After the completion of the training process, the MAB-ensemble technique underwent testing using the test dataset from the TuSimple dataset, which comprises 667 road lane images. To comprehensively evaluate performance, the test set images are categorized into three groups based on environmental and road conditions. Specifically, 432 images are categorized as daytime, 172 images as nighttime, and the remaining 63 images as abnormal road conditions. The hardware used for all experiments is an NVIDIA A100 GPU. Subsequently, the experimental results of the MAB-ensemble technique on the test set are analyzed and discussed in the subsequent subsection. This examination aims to provide insights into the efficacy of the proposed technique in addressing lane detection challenges across varied environmental and road conditions.

**Fig. 5:**
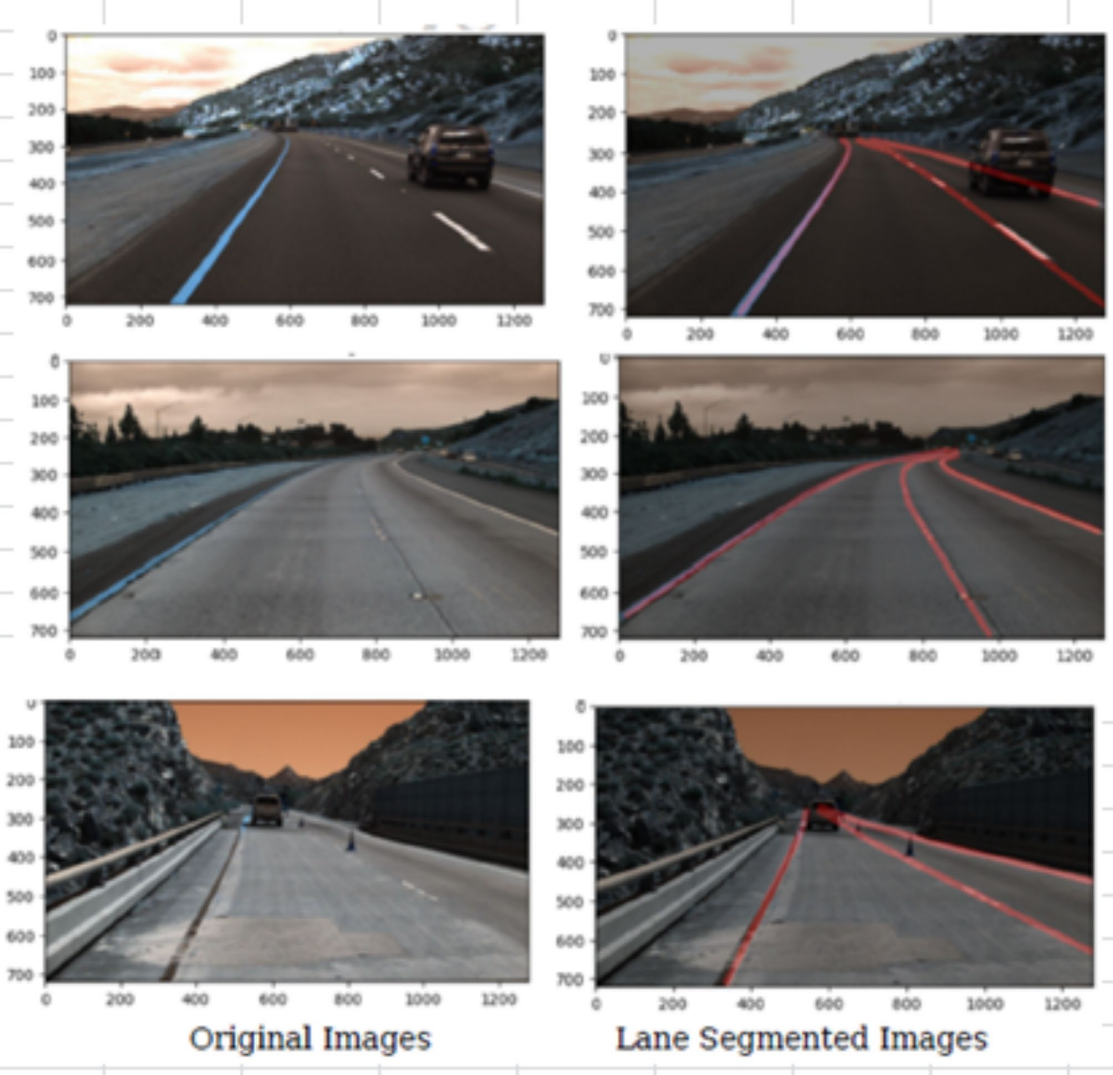
Sample lane predictions of the proposed MAB-ensemble technique.

The performance assessment of the proposed MAB-ensemble technique evaluates its efficacy in lane recognition across varying road conditions, including daytime, nighttime, and abnormal scenarios, utilizing the test set images. The sample input images and the segmented outputs by the proposed MAB-ensemble technique are shown in Fig. 5.

To measure the performance of the proposed and existing lane detection techniques, standard performance metrics for segmentation tasks, namely segmentation accuracy, RMSE, IoU and Dice Coefficient (or F1 Score) are employed. Segmentation accuracy measures the proportion of correctly identified lane pixels compared to the total number of lane pixels in the ground truth. The RMSE quantifies the average deviation between predicted and ground truth lane positions, which provides insight into the precision of lane detection. IoU measures the overlap between the predicted segmentation mask and the ground truth mask, indicating the quality of the segmentation. The dice coefficient measures the similarity between the predicted and ground truth segmentation masks.

The Accuracy, RMSE, IoU and Dice Coefficient metrics for lane segmentation can be calculated as follows:

3 $$\:\text{A}\text{c}\text{c}\text{u}\text{r}\text{a}\text{c}\text{y}=\:\frac{\text{T}\text{P}+\text{T}\text{N}}{\text{T}\text{P}+\text{T}\text{N}+\text{F}\text{P}+\text{F}\text{N}}\:\times\:100$$

4 $$\:\text{R}\text{M}\text{S}\text{E}=\:\sqrt{\frac{{{\sum\:}_{\text{i}=1}^{\text{n}}\left({\text{y}}_{\text{i}}-\:\overline{{\text{y}}_{\text{i}}}\right)}^{2}}{\text{n}}}$$

5 $$\:\text{I}\text{o}\text{U}=\:\frac{\text{T}\text{P}}{\text{T}\text{P}+\text{F}\text{P}+\text{F}\text{N}}\:\times\:100$$

6 $$\:\text{D}\text{i}\text{c}\text{e}\:\text{C}\text{o}\text{e}\text{f}\text{f}\text{i}\text{c}\text{i}\text{e}\text{n}\text{t}=\:\frac{2\:\times\:\:\left|\text{A}\:\cap\:\text{B}\right|}{\left|\text{A}\right|+\:\left|\text{B}\right|}\:\times\:100$$

Where the TP (True Positives) represents correctly identified lane pixels, the TN (True Negatives) denotes correctly identified background pixels, the FP (False Positives) indicates incorrectly classified lane pixels as background, the FN (False Negatives) signifies incorrectly classified background pixels as lane, the y_i_​ represents the ground truth lane position, the $\:\overline{{\text{y}}_{\text{i}}}$ denotes the predicted lane position, n denotes the total number of samples A is the set of pixels in the predicted mask and B is the set of pixels in the ground truth mask.

The proposed MAB-ensemble technique achieved an overall accuracy of 90.28%, an RMSE of 13.92, an IoU of 91.51%, and a Dice Coefficient of 92.73%. Subsequently, the performance of the MAB-ensemble technique was compared with existing lane detection techniques under diverse road conditions. The accuracy, RMSE, IoU and Dice Coefficient serve as primary performance metrics across three distinct road conditions: daytime, nighttime, and abnormal road conditions. Each condition is assessed using a dedicated subset of the test dataset.

Initially, the segmentation techniques are assessed under daytime conditions by utilizing 432 images from the test dataset. The performance comparison results of the proposed MAB-ensemble technique and the existing techniques on daytime conditions are shown in Table 3. The proposed MAB-ensemble technique achieves an accuracy of 98.74%, RMSE of 11.99, IoU of 98.02% and dice coefficient of 99.45% outperforming the existing techniques namely, ENet, PINet, ResNet-50, ResNet-101, SqueezeNet, and VGG16Net.

**Table 3:** Performance comparison in Daytime road conditions.

Segmentation Model	Accuracy	RMSE	IoU	Dice Coefficient
Enet	92.33	14.62	94.39	90.41
MAB-Ensemble	98.74	11.99	98.02	99.45
PINet	94.87	14.52	97.01	93.05
ResNet101	91.83	14.71	93.88	89.82
ResNet50	90.77	15.18	92.79	88.29
SqueezeNet	91.38	14.83	93.42	89.25
VGG16Net	87.43	15.48	89.35	84.65

Subsequently, segmentation techniques are evaluated under nighttime conditions using 172 images from the test dataset, where the proposed technique demonstrates superior performance with an accuracy of 92.71%, higher than existing techniques, with accuracy, RMSE, IoU, and Dice coefficient comparisons between the proposed MAB-ensemble technique and existing methods illustrated in Table 4.

**Table 4:** Performance comparison in nighttime road conditions.

Segmentation Model	Accuracy	RMSE	IoU	Dice Coefficient
ENet	81.67	23.32	82.95	83.67
MAB-Ensemble	92.71	13.75	94.24	95.28
PINet	80.07	23.68	81.31	81.71
ResNet101	77.43	23.87	78.61	78.88
ResNet50	76.38	24.28	77.54	77.42
SqueezeNet	83.74	22.24	85.07	86.82
VGG16Net	72.94	25.63	74.02	72.63

Succeeding, the segmentation techniques are analyzed under abnormal road conditions using 63 images from the test dataset, where the proposed technique continues to exhibit superior performance with an accuracy of 79.38%, outperforming existing techniques, and the performance comparison results between the proposed MAB-ensemble technique and existing methods are presented in Table 5.

**Table 5:** Performance comparison in abnormal road conditions.

Segmentation Model	Accuracy	RMSE	IoU	Dice Coefficient
ENet	65.71	19.77	68.3	69.38
MAB-Ensemble	79.38	16.03	82.26	83.47
PINet	63.78	20.37	66.33	67.39
ResNet101	60.57	22.71	63.05	64.08
ResNet50	58.16	23.06	60.59	61.59
SqueezeNet	62.49	21.21	65.01	66.06
VGG16Net	68.43	19.37	71.08	72.18

Finally, the segmentation techniques are assessed on the entire test dataset, comprising 667 images, where the proposed MAB-ensemble technique maintains its superior performance with an accuracy of 90.28%, outperforming existing techniques, as shown in the performance comparison results in Table 6.

**Table 6:** Performance comparison in the entire test data.

Segmentation Model	Accuracy	RMSE	IoU	Dice Coefficient
ENet	79.9	19.24	81.88	81.15
MAB-Ensemble	90.28	13.92	91.51	92.73
PINet	79.57	19.52	81.55	80.72
ResNet101	76.61	20.43	78.51	77.59
ResNet50	75.1	20.84	76.97	75.77
SqueezeNet	79.2	19.43	81.17	80.71
VGG16Net	76.27	20.16	78.15	76.49

The analysis revealed that the performance of existing lane detection techniques varied across different road conditions, highlighting the need for more adaptive and robust approaches. In contrast, the proposed MAB-Ensemble technique outperformed existing methods by dynamically selecting the most suitable model based on the prevailing environmental and road conditions. This superior performance is due to the technique’s ability to leverage the advantages of multiple CNN models, including ENet, PINet, ResNet-50, ResNet-101, SqueezeNet, and VGG16Net, each optimized for specific scenarios such as varied lighting and road conditions.

Table 7 shows a comparative analysis of the performance of the MAB-Ensemble technique against several widely used ensemble techniques, including Majority Voting, Random Forest, Stacking, and Weighted Averaging, in terms of key evaluation metrics for lane segmentation. It highlights the accuracy, Root Mean Squared Error (RMSE), Intersection over Union (IoU), and Dice Coefficient for each model.

**Table 7:** Performance comparison with ensemble techniques.

Segmentation Model	Accuracy	RMSE	IoU	Dice Coefficient
MAB-Ensemble	90.28	13.92	91.51	92.73
Majority Voting	84.5	17.28	85.34	85.10
Random Forest	85.3	16.78	86.12	85.78
Stacking	87.1	15.48	88.22	87.85
Weighted Averaging	83.9	17.68	84.78	84.47

The result demonstrates the relative strengths and weaknesses of the different ensemble approaches in addressing the lane detection task. It highlights how MAB-Ensemble outperforms traditional ensemble methods, underscoring the effectiveness of dynamic model selection in adapting to changing environmental and road conditions for improved performance.

Table 8 shows the comparison of Convergence Time and Inference Time for various segmentation models, including the proposed MAB-Ensemble and other ensemble techniques. Convergence time represents the time taken by the model to reach optimal performance during training, while inference time indicates the average time required to process a single test instance once the model is trained.

**Table 8:** Convergence and inference time comparison.

Segmentation Model	Convergence Time (Seconds)	Inference Time (Seconds)
ENet	453.5	0.48
MAB-Ensemble	557.2	0.51
Majority Voting	586.2	0.49
PINet	431.1	0.47
Random Forest	627.1	0.54
ResNet101	702.3	0.53
ResNet50	686.4	0.54
SqueezeNet	424.2	0.46
Stacking	609.5	0.56
VGG16Net	652.7	0.55
Weighted Averaging	575.8	0.58

The MAB-Ensemble model takes 557.2 s to converge, which is higher than some other models such as ENet (453.5 s) and PINet (431.1 s), but still within a reasonable range compared to others like ResNet101 (702.3 s) and ResNet50 (686.4 s). As for inference time, MAB-Ensemble takes 0.51 s, which is competitive with other models such as Majority Voting (0.49 s) and SqueezeNet (0.46 s), and still faster than models like Stacking (0.56 s) and Weighted Averaging (0.58 s).

The MAB-Ensemble dynamically adjusts model selection by employing Multi-Armed Bandit optimization with Thompson sampling. It treats the performance of each CNN as a reward signal. This enables the system to favour models that yield higher accuracy under specific conditions. The approach reduces overfitting, improves generalization, and adapts to new environmental factors. As a result, the MAB-Ensemble achieves robust accuracy across diverse conditions. It reaches an overall accuracy of 90.28% on the TuSimple dataset. The technique excels in specific scenarios, such as daytime (98.74%), nighttime (92.71%), and abnormal conditions (79.38%). Furthermore, the flexibility of the MAB-Ensemble framework allows for the incorporation of future CNN advancements or adjustments to model selection criteria. This ensures scalability and adaptability to real-world complexities in autonomous driving scenarios. These accuracy results highlight the efficacy of the MAB-Ensemble technique in addressing lane detection challenges across various real-world scenarios and advancing the field of autonomous driving systems and computer vision applications.

The MAB-Ensemble technique has certain limitations while demonstrating significant improvements in lane detection. One primary challenge is the potential delay in convergence, especially in highly volatile environments, which may impact real-time performance. The balance between exploration and exploitation in the Multi-Armed Bandit approach can result in suboptimal model selection and increased computational overhead if not finely tuned. Additionally, the dynamic model selection’s effectiveness depends on having a sufficiently large pool of models to choose from, which could limit its performance when fewer models are available. Although the technique reduces latency, the computational cost associated with dynamic model switching could present challenges in resource-constrained settings. Despite these limitations, the MAB-Ensemble approach offers a robust and adaptive solution, particularly in environments that experience dynamic and varied conditions.

## Conclusion

Lane detection and tracking are predominant for the safety and efficiency of autonomous vehicles. This study proposes an innovative ensemble learning approach, termed the MAB-Ensemble technique, employing multi-armed bandit optimization with Thompson sampling to detect lanes in road images, thereby aiding autonomous vehicles. Utilizing popular CNNs such as ENet, PINet, ResNet-50, ResNet-101, SqueezeNet, and VGG16Net, segmentation models are developed by adapting the final layers. The TuSimple dataset was used to train and test the proposed MAB-Ensemble technique and individual CNNs. While individual CNN models exhibit proficiency in specific road conditions, the MAB-Ensemble technique dynamically selects the most suitable model for lane segmentation based on prevailing environmental factors. By engaging in environment interaction through action selection and rewards, the segmentation accuracy serves as the reward signal. The ensemble technique optimizes the performance across varied conditions and achieves an impressive overall accuracy of 90.28% in the dataset of 667 road images. Furthermore, its accuracy in daytime, nighttime, and abnormal road conditions reached 98.74%, 92.71%, and 79.38%, respectively. Notably, the MAB-Ensemble technique demonstrated superior performance across all road conditions compared to individual CNN models and traditional ensemble techniques. There are opportunities for further improvement while the MAB-Ensemble model demonstrates strong performance. Future work could focus on optimizing convergence time through more efficient techniques and simplifying the model selection process. Exploring hybrid reward systems and advanced exploration strategies could enhance adaptability in dynamic environments. Additionally, incorporating lightweight models or pruning methods could reduce computational overhead, ensuring the model’s effectiveness in real-time, resource-constrained applications. These enhancements would further strengthen the MAB-Ensemble’s practical applicability, especially in autonomous driving scenarios.

## Data Availability

The data that support the findings of this study are available on request from the corresponding author.
